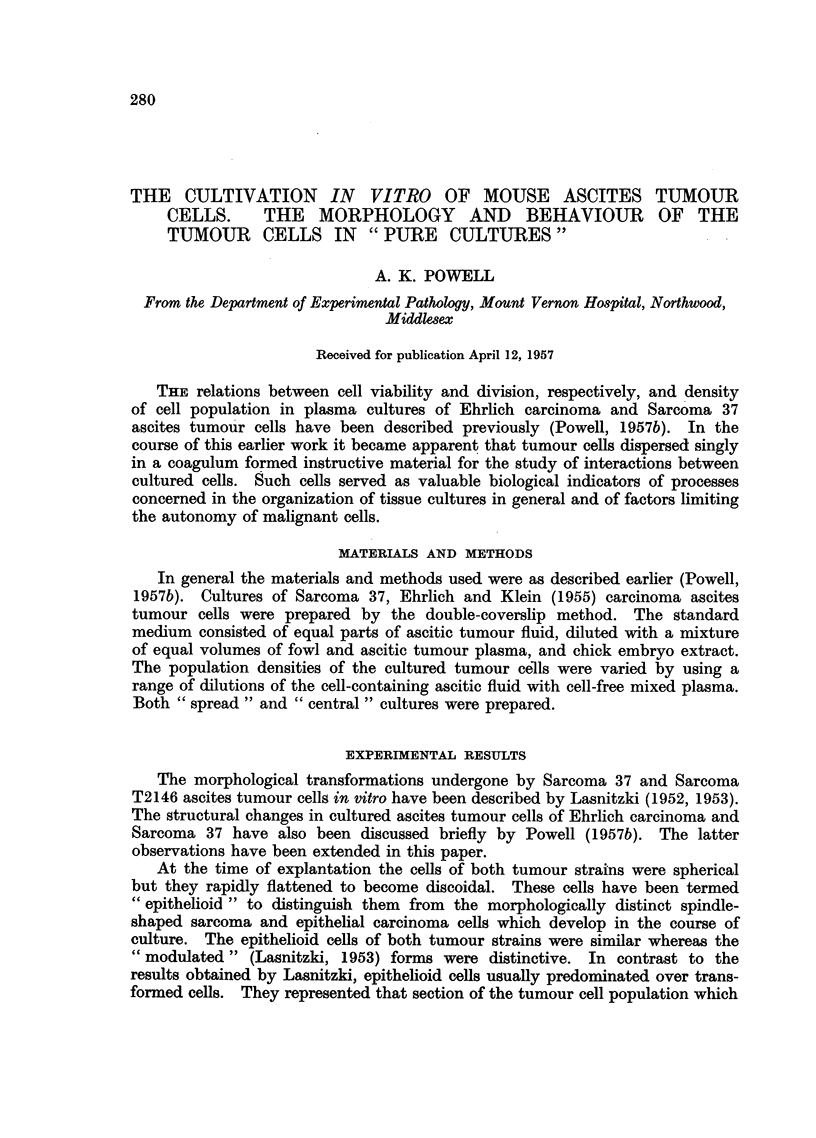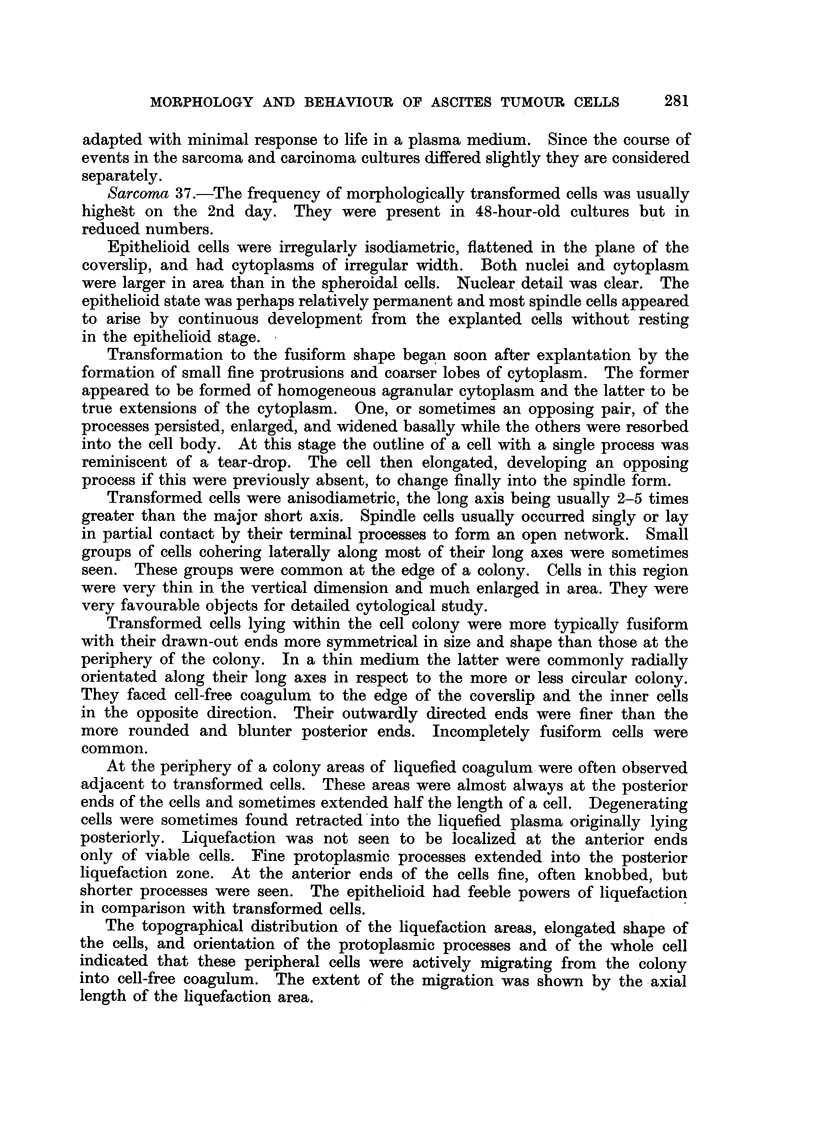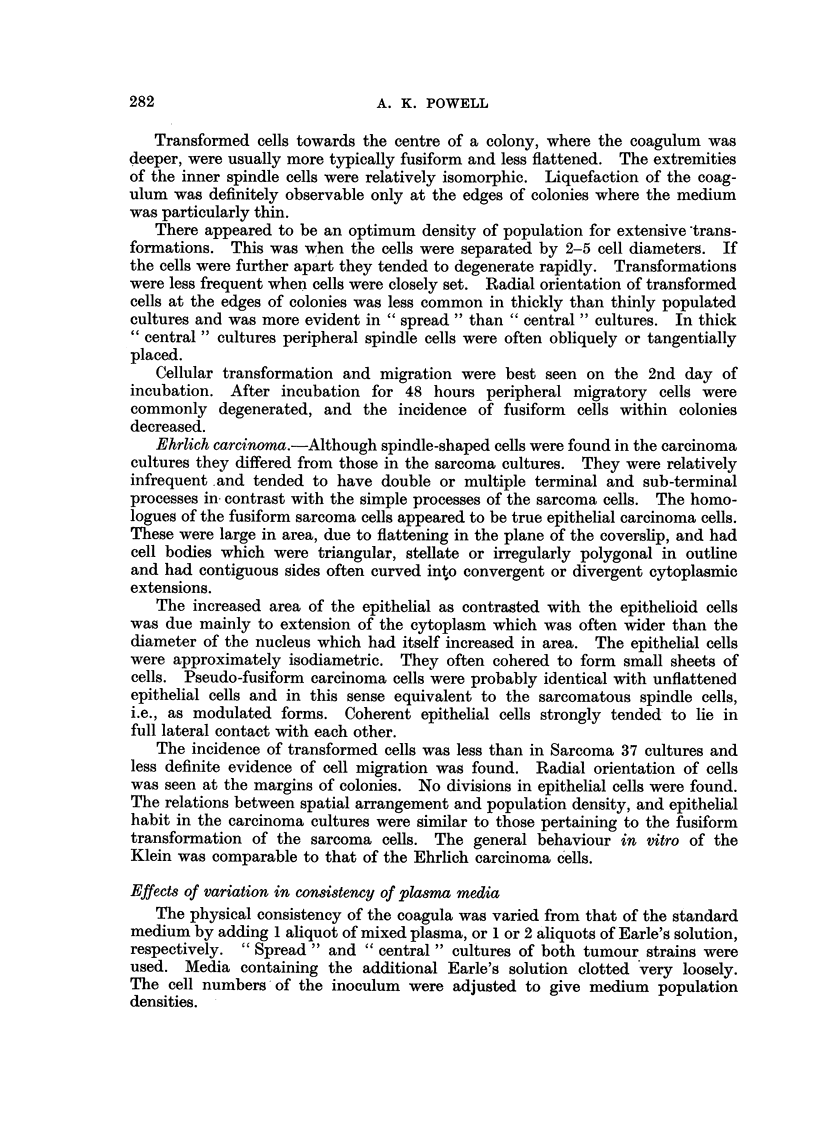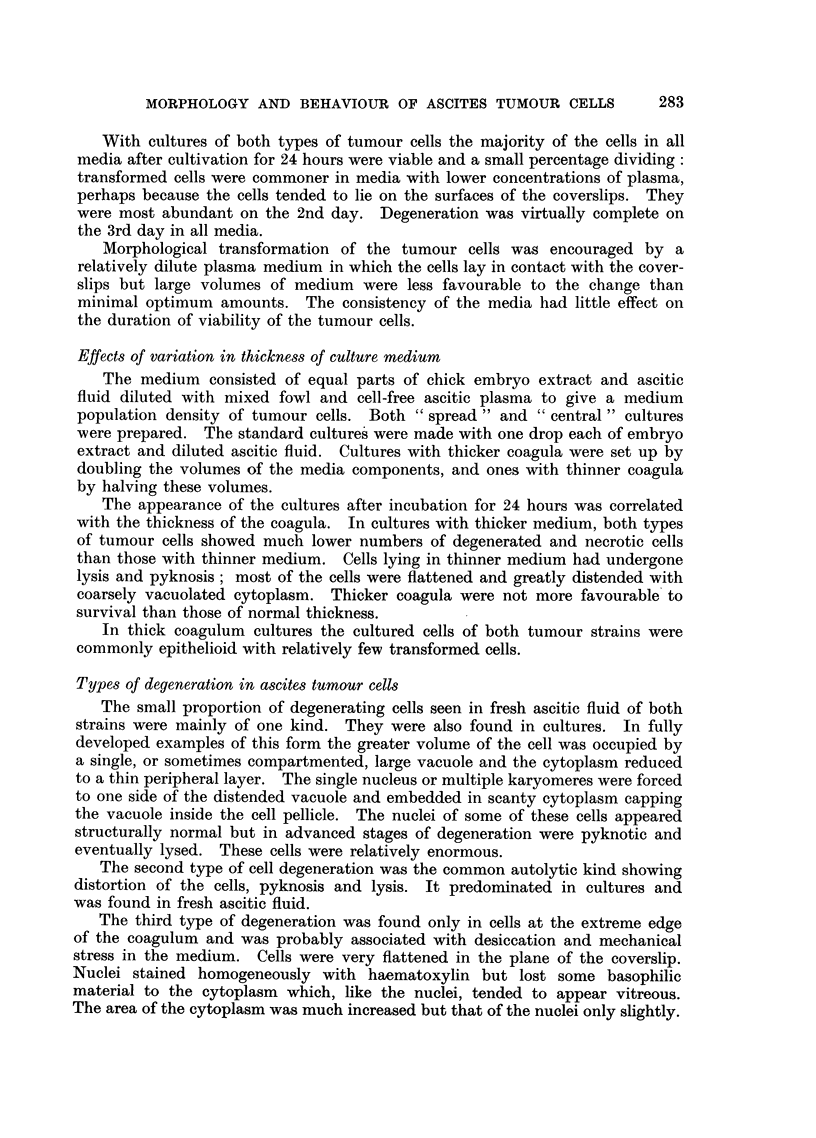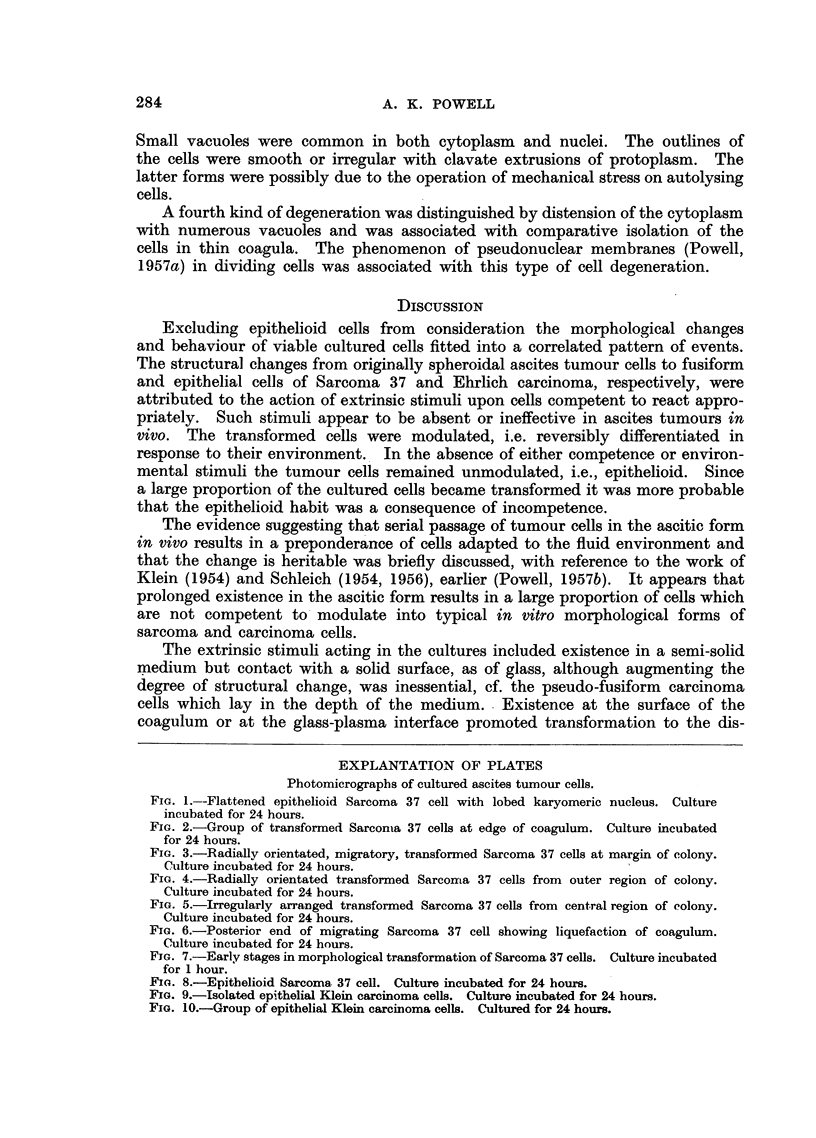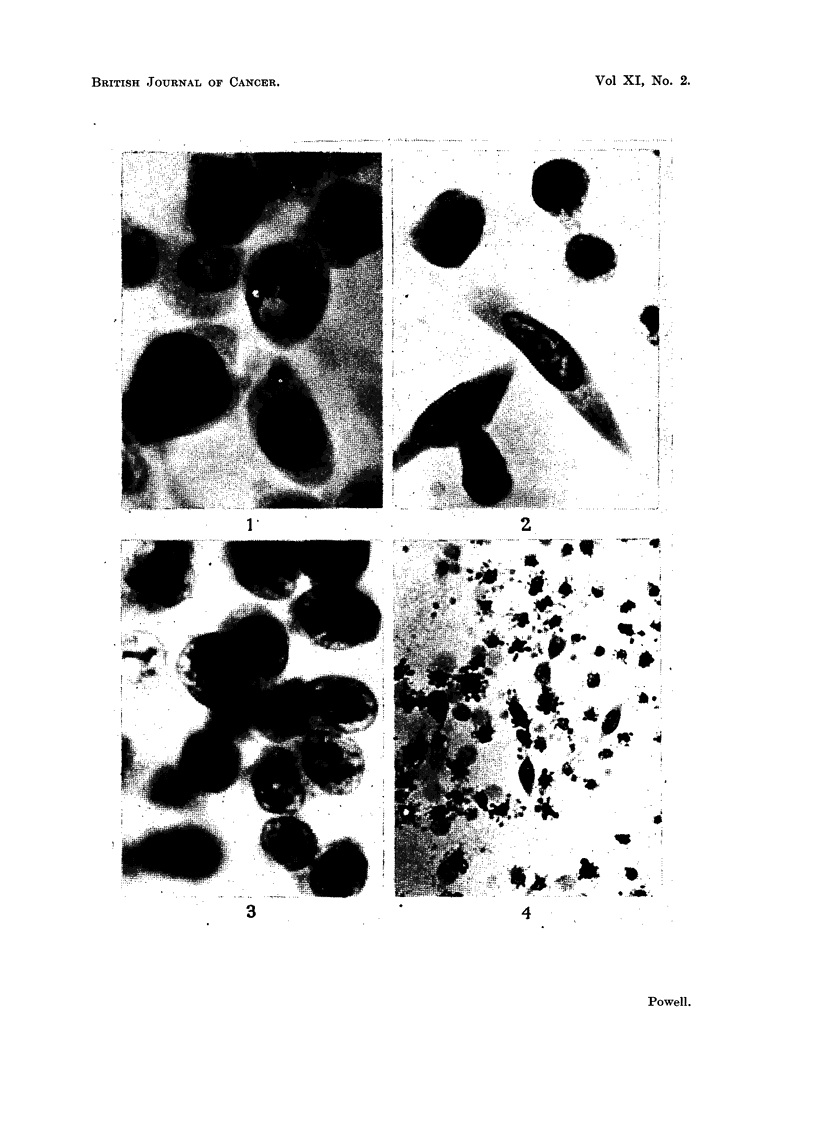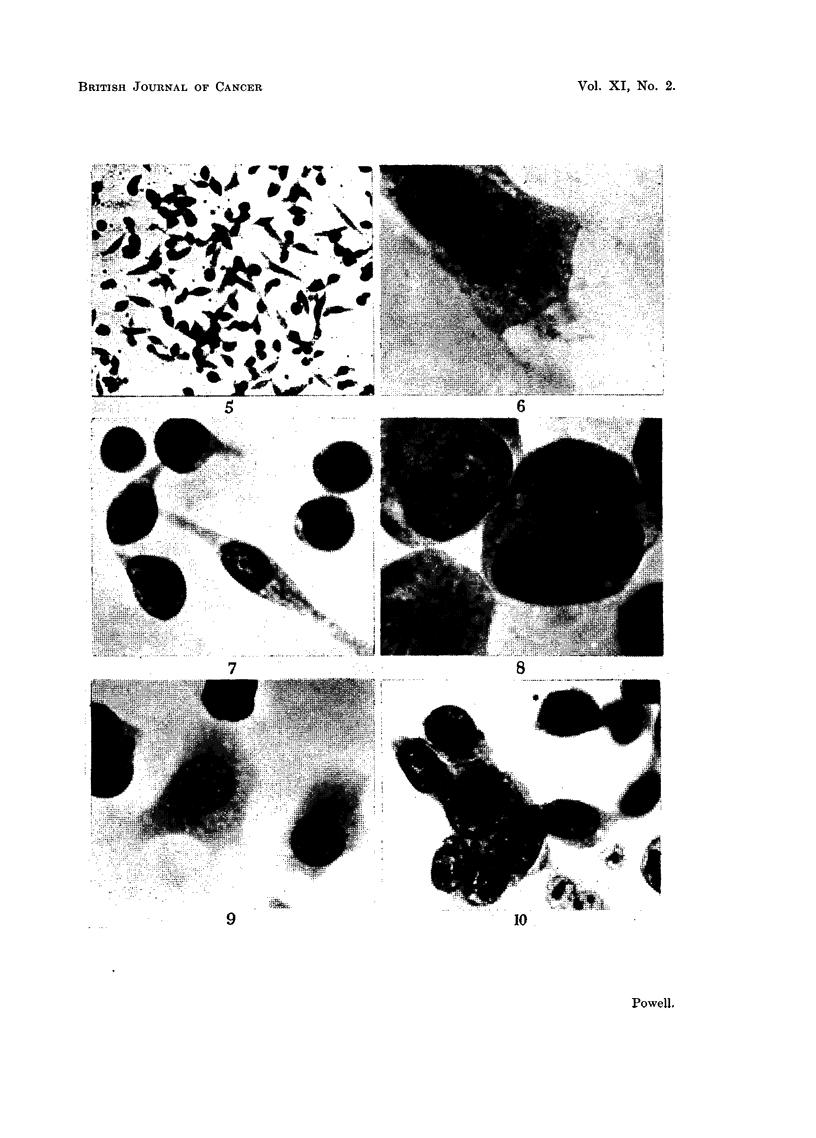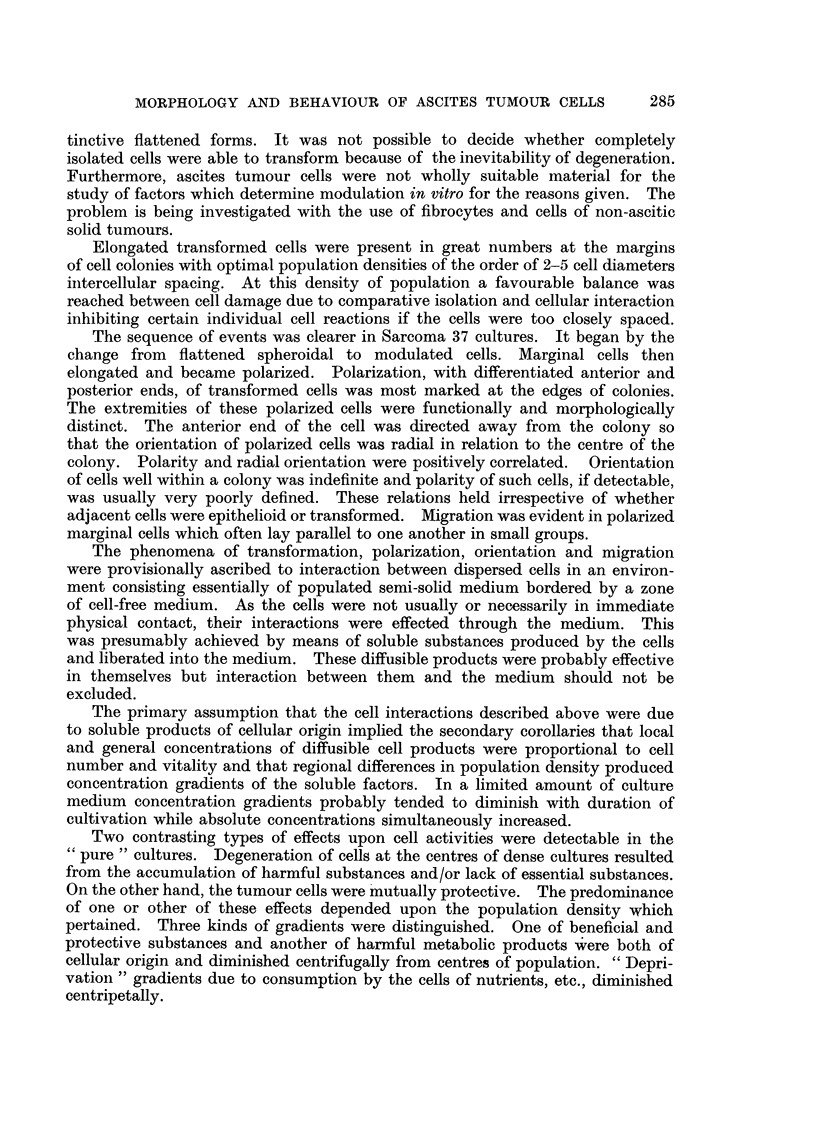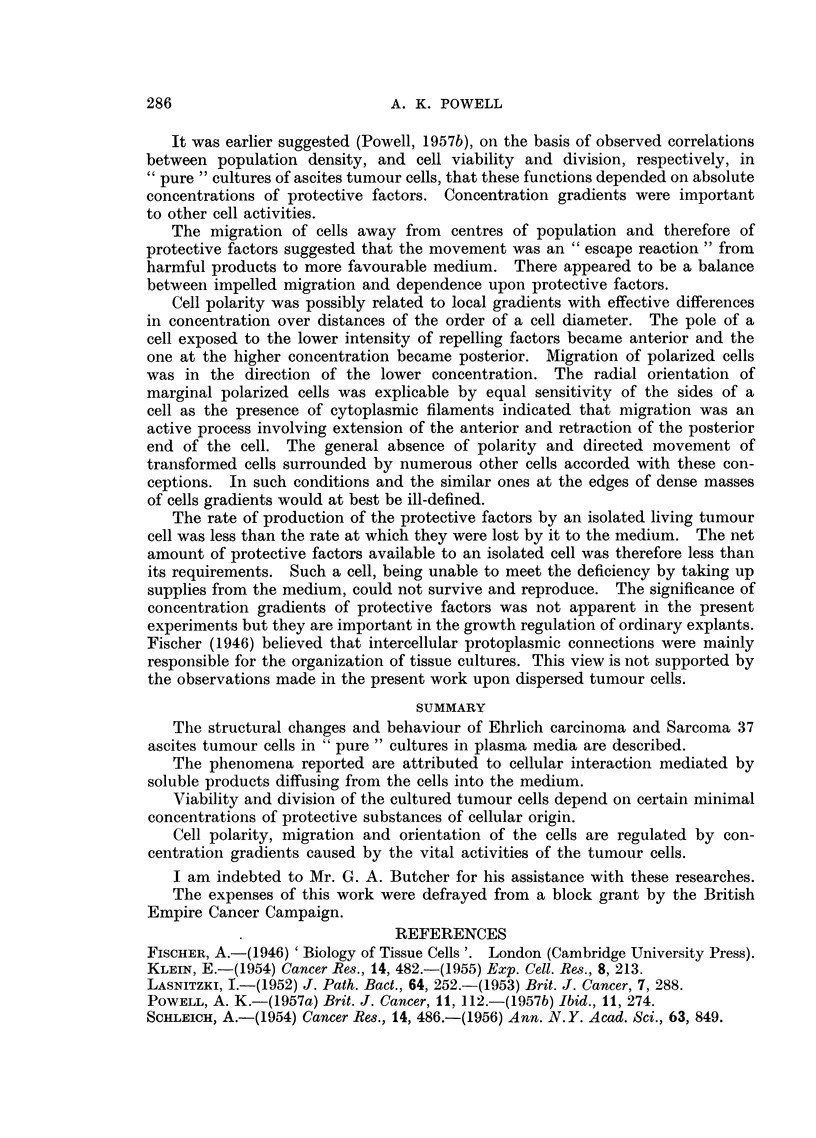# The Cultivation In Vitro of Mouse Ascites Tumour Cells. The Morphology and Behaviour of the Tumour Cells in “Pure Cultures”

**DOI:** 10.1038/bjc.1957.36

**Published:** 1957-06

**Authors:** A. K. Powell

## Abstract

**Images:**


### THE CULTIVATION *IN VITRO* OF MOUSE ASCITES TUMOUR CELLS. THE MORPHOLOGY AND BEHAVIOUR OF THE TUMOUR CELLS IN "PURE CULTURES"

A. K. POWELL

*From the Department of Experimental Pathology, Mount Vernon Hospital, Northwood, Middlesex*

Received for publication April 12, 1957

THE relations between cell viability and division, respectively, and density of cell population in plasma cultures of Ehrlich carcinoma and Sarcoma 37 ascites tumour cells have been described previously (Powell, 1957*b*). In the course of this earlier work it became apparent that tumour cells dispersed singly in a coagulum formed instructive material for the study of interactions between cultured cells. Such cells served as valuable biological indicators of processes concerned in the organization of tissue cultures in general and of factors limiting the autonomy of malignant cells.

### MATERIALS AND METHODS

In general the materials and methods used were as described earlier (Powell, 1957*b*). Cultures of Sarcoma 37, Ehrlich and Klein (1955) carcinoma ascites tumour cells were prepared by the double-coverslip method. The standard medium consisted of equal parts of ascitic tumour fluid, diluted with a mixture of equal volumes of fowl and ascitic tumour plasma, and chick embryo extract. The population densities of the cultured tumour cells were varied by using a range of dilutions of the cell-containing ascitic fluid with cell-free mixed plasma. Both "spread" and "central" cultures were prepared.

### EXPERIMENTAL RESULTS

The morphological transformations undergone by Sarcoma 37 and Sarcoma T2146 ascites tumour cells *in vitro* have been described by Lasnitzki (1952, 1953). The structural changes in cultured ascites tumour cells of Ehrlich carcinoma and Sarcoma 37 have also been discussed briefly by Powell (1957*b*). The latter observations have been extended in this paper.

At the time of explantation the cells of both tumour strains were spherical but they rapidly flattened to become discoidal. These cells have been termed "epithelioid" to distinguish them from the morphologically distinct spindle-shaped sarcoma and epithelial carcinoma cells which develop in the course of culture. The epithelioid cells of both tumour strains were similar whereas the "modulated" (Lasnitzki, 1953) forms were distinctive. In contrast to the results obtained by Lasnitzki, epithelioid cells usually predominated over transformed cells. They represented that section of the tumour cell population which adapted with minimal response to life in a plasma medium. Since the course of events in the sarcoma and carcinoma cultures differed slightly they are considered separately.

*Sarcoma* 37.—The frequency of morphologically transformed cells was usually highest on the 2nd day. They were present in 48-hour-old cultures but in reduced numbers.

Epithelioid cells were irregularly isodiametric, flattened in the plane of the coverslip, and had cytoplasms of irregular width. Both nuclei and cytoplasm were larger in area than in the spheroidal cells. Nuclear detail was clear. The epithelioid state was perhaps relatively permanent and most spindle cells appeared to arise by continuous development from the explanted cells without resting in the epithelioid stage.

Transformation to the fusiform shape began soon after explantation by the formation of small fine protrusions and coarser lobes of cytoplasm. The former appeared to be formed of homogeneous agranular cytoplasm and the latter to be true extensions of the cytoplasm. One, or sometimes an opposing pair, of the processes persisted, enlarged, and widened basally while the others were resorbed into the cell body. At this stage the outline of a cell with a single process was reminiscent of a tear-drop. The cell then elongated, developing an opposing process if this were previously absent, to change finally into the spindle form.

Transformed cells were anisodiametric, the long axis being usually 2–5 times greater than the major short axis. Spindle cells usually occurred singly or lay in partial contact by their terminal processes to form an open network. Small groups of cells cohering laterally along most of their long axes were sometimes seen. These groups were common at the edge of a colony. Cells in this region were very thin in the vertical dimension and much enlarged in area. They were very favourable objects for detailed cytological study.

Transformed cells lying within the cell colony were more typically fusiform with their drawn-out ends more symmetrical in size and shape than those at the periphery of the colony. In a thin medium the latter were commonly radially orientated along their long axes in respect to the more or less circular colony. They faced cell-free coagulum to the edge of the coverslip and the inner cells in the opposite direction. Their outwardly directed ends were finer than the more rounded and blunter posterior ends. Incompletely fusiform cells were common.

At the periphery of a colony areas of liquefied coagulum were often observed adjacent to transformed cells. These areas were almost always at the posterior ends of the cells and sometimes extended half the length of a cell. Degenerating cells were sometimes found retracted into the liquefied plasma originally lying posteriorly. Liquefaction was not seen to be localized at the anterior ends only of viable cells. Fine protoplasmic processes extended into the posterior liquefaction zone. At the anterior ends of the cells fine, often knobbed, but shorter processes were seen. The epithelioid had feeble powers of liquefaction in comparison with transformed cells.

The topographical distribution of the liquefaction areas, elongated shape of the cells, and orientation of the protoplasmic processes and of the whole cell indicated that these peripheral cells were actively migrating from the colony into cell-free coagulum. The extent of the migration was shown by the axial length of the liquefaction area.

Transformed cells towards the centre of a colony, where the coagulum was deeper, were usually more typically fusiform and less flattened. The extremities of the inner spindle cells were relatively isomorphic. Liquefaction of the coagulum was definitely observable only at the edges of colonies where the medium was particularly thin.

There appeared to be an optimum density of population for extensive transformations. This was when the cells were separated by 2–5 cell diameters. If the cells were further apart they tended to degenerate rapidly. Transformations were less frequent when cells were closely set. Radial orientation of transformed cells at the edges of colonies was less common in thickly than thinly populated cultures and was more evident in " spread " than " central " cultures. In thick " central " cultures peripheral spindle cells were often obliquely or tangentially placed.

Cellular transformation and migration were best seen on the 2nd day of incubation. After incubation for 48 hours peripheral migratory cells were commonly degenerated, and the incidence of fusiform cells within colonies decreased.

*Ehrlich carcinoma.*—Although spindle-shaped cells were found in the carcinoma cultures they differed from those in the sarcoma cultures. They were relatively infrequent and tended to have double or multiple terminal and sub-terminal processes in contrast with the simple processes of the sarcoma cells. The homologues of the fusiform sarcoma cells appeared to be true epithelial carcinoma cells. These were large in area, due to flattening in the plane of the coverslip, and had cell bodies which were triangular, stellate or irregularly polygonal in outline and had contiguous sides often curved into convergent or divergent cytoplasmic extensions.

The increased area of the epithelial as contrasted with the epithelioid cells was due mainly to extension of the cytoplasm which was often wider than the diameter of the nucleus which had itself increased in area. The epithelial cells were approximately isodiametric. They often cohered to form small sheets of cells. Pseudo-fusiform carcinoma cells were probably identical with unflattened epithelial cells and in this sense equivalent to the sarcomatous spindle cells, i.e., as modulated forms. Coherent epithelial cells strongly tended to lie in full lateral contact with each other.

The incidence of transformed cells was less than in Sarcoma 37 cultures and less definite evidence of cell migration was found. Radial orientation of cells was seen at the margins of colonies. No divisions in epithelial cells were found. The relations between spatial arrangement and population density, and epithelial habit in the carcinoma cultures were similar to those pertaining to the fusiform transformation of the sarcoma cells. The general behaviour *in vitro* of the Klein was comparable to that of the Ehrlich carcinoma cells.

### *Effects of variation in consistency of plasma media*

The physical consistency of the coagula was varied from that of the standard medium by adding 1 aliquot of mixed plasma, or 1 or 2 aliquots of Earle's solution, respectively. " Spread " and " central " cultures of both tumour strains were used. Media containing the additional Earle's solution clotted very loosely. The cell numbers of the inoculum were adjusted to give medium population densities.

With cultures of both types of tumour cells the majority of the cells in all media after cultivation for 24 hours were viable and a small percentage dividing : transformed cells were commoner in media with lower concentrations of plasma, perhaps because the cells tended to lie on the surfaces of the coverslips. They were most abundant on the 2nd day. Degeneration was virtually complete on the 3rd day in all media.

Morphological transformation of the tumour cells was encouraged by a relatively dilute plasma medium in which the cells lay in contact with the coverslips but large volumes of medium were less favourable to the change than minimal optimum amounts. The consistency of the media had little effect on the duration of viability of the tumour cells.

*Effects of variation in thickness of culture medium*

The medium consisted of equal parts of chick embryo extract and ascitic fluid diluted with mixed fowl and cell-free ascitic plasma to give a medium population density of tumour cells. Both " spread " and " central " cultures were prepared. The standard cultures were made with one drop each of embryo extract and diluted ascitic fluid. Cultures with thicker coagula were set up by doubling the volumes of the media components, and ones with thinner coagula by halving these volumes.

The appearance of the cultures after incubation for 24 hours was correlated with the thickness of the coagula. In cultures with thicker medium, both types of tumour cells showed much lower numbers of degenerated and necrotic cells than those with thinner medium. Cells lying in thinner medium had undergone lysis and pyknosis ; most of the cells were flattened and greatly distended with coarsely vacuolated cytoplasm. Thicker coagula were not more favourable to survival than those of normal thickness.

In thick coagulum cultures the cultured cells of both tumour strains were commonly epithelioid with relatively few transformed cells.

*Types of degeneration in ascites tumour cells*

The small proportion of degenerating cells seen in fresh ascitic fluid of both strains were mainly of one kind. They were also found in cultures. In fully developed examples of this form the greater volume of the cell was occupied by a single, or sometimes compartmented, large vacuole and the cytoplasm reduced to a thin peripheral layer. The single nucleus or multiple karyomeres were forced to one side of the distended vacuole and embedded in scanty cytoplasm capping the vacuole inside the cell pellicle. The nuclei of some of these cells appeared structurally normal but in advanced stages of degeneration were pyknotic and eventually lysed. These cells were relatively enormous.

The second type of cell degeneration was the common autolytic kind showing distortion of the cells, pyknosis and lysis. It predominated in cultures and was found in fresh ascitic fluid.

The third type of degeneration was found only in cells at the extreme edge of the coagulum and was probably associated with desiccation and mechanical stress in the medium. Cells were very flattened in the plane of the coverslip. Nuclei stained homogeneously with haematoxylin but lost some basophilic material to the cytoplasm which, like the nuclei, tended to appear vitreous. The area of the cytoplasm was much increased but that of the nuclei only slightly.

Small vacuoles were common in both cytoplasm and nuclei. The outlines of the cells were smooth or irregular with clavate extrusions of protoplasm. The latter forms were possibly due to the operation of mechanical stress on autolysing cells.

A fourth kind of degeneration was distinguished by distension of the cytoplasm with numerous vacuoles and was associated with comparative isolation of the cells in thin coagula. The phenomenon of pseudonuclear membranes (Powell, 1957*a*) in dividing cells was associated with this type of cell degeneration.

### Discussion

Excluding epithelioid cells from consideration the morphological changes and behaviour of viable cultured cells fitted into a correlated pattern of events. The structural changes from originally spheroidal ascites tumour cells to fusiform and epithelial cells of Sarcoma 37 and Ehrlich carcinoma, respectively, were attributed to the action of extrinsic stimuli upon cells competent to react appropriately. Such stimuli appear to be absent or ineffective in ascites tumours *in vivo*. The transformed cells were modulated, i.e. reversibly differentiated in response to their environment. In the absence of either competence or environmental stimuli the tumour cells remained unmodulated, i.e., epithelioid. Since a large proportion of the cultured cells became transformed it was more probable that the epithelioid habit was a consequence of incompetence.

The evidence suggesting that serial passage of tumour cells in the ascitic form *in vivo* results in a preponderance of cells adapted to the fluid environment and that the change is heritable was briefly discussed, with reference to the work of Klein (1954) and Schleich (1954, 1956), earlier (Powell, 1957*b*). It appears that prolonged existence in the ascitic form results in a large proportion of cells which are not competent to modulate into typical *in vitro* morphological forms of sarcoma and carcinoma cells.

The extrinsic stimuli acting in the cultures included existence in a semi-solid medium but contact with a solid surface, as of glass, although augmenting the degree of structural change, was inessential, cf. the pseudo-fusiform carcinoma cells which lay in the depth of the medium. Existence at the surface of the coagulum or at the glass-plasma interface promoted transformation to the dis-

---

### EXPLANATION OF PLATES

Photomicrographs of cultured ascites tumour cells.

Fig. 1.—Flattened epithelioid Sarcoma 37 cell with lobed karyomeric nucleus. Culture incubated for 24 hours.

Fig. 2.—Group of transformed Sarcoma 37 cells at edge of coagulum. Culture incubated for 24 hours.

Fig. 3.—Radially orientated, migratory, transformed Sarcoma 37 cells at margin of colony. Culture incubated for 24 hours.

Fig. 4.—Radially orientated transformed Sarcoma 37 cells from outer region of colony. Culture incubated for 24 hours.

Fig. 5.—Irregularly arranged transformed Sarcoma 37 cells from central region of colony. Culture incubated for 24 hours.

Fig. 6.—Posterior end of migrating Sarcoma 37 cell showing liquefaction of coagulum. Culture incubated for 24 hours.

Fig. 7.—Early stages in morphological transformation of Sarcoma 37 cells. Culture incubated for 1 hour.

Fig. 8.—Epithelioid Sarcoma 37 cell. Culture incubated for 24 hours.

Fig. 9.—Isolated epithelial Klein carcinoma cells. Culture incubated for 24 hours.

Fig. 10.—Group of epithelial Klein carcinoma cells. Cultured for 24 hours.

Powell.

5 6 7 8 9 10

Powell.

tinctive flattened forms. It was not possible to decide whether completely isolated cells were able to transform because of the inevitability of degeneration. Furthermore, ascites tumour cells were not wholly suitable material for the study of factors which determine modulation *in vitro* for the reasons given. The problem is being investigated with the use of fibrocytes and cells of non-ascitic solid tumours.

Elongated transformed cells were present in great numbers at the margins of cell colonies with optimal population densities of the order of 2–5 cell diameters intercellular spacing. At this density of population a favourable balance was reached between cell damage due to comparative isolation and cellular interaction inhibiting certain individual cell reactions if the cells were too closely spaced.

The sequence of events was clearer in Sarcoma 37 cultures. It began by the change from flattened spheroidal to modulated cells. Marginal cells then elongated and became polarized. Polarization, with differentiated anterior and posterior ends, of transformed cells was most marked at the edges of colonies. The extremities of these polarized cells were functionally and morphologically distinct. The anterior end of the cell was directed away from the colony so that the orientation of polarized cells was radial in relation to the centre of the colony. Polarity and radial orientation were positively correlated. Orientation of cells well within a colony was indefinite and polarity of such cells, if detectable, was usually very poorly defined. These relations held irrespective of whether adjacent cells were epithelioid or transformed. Migration was evident in polarized marginal cells which often lay parallel to one another in small groups.

The phenomena of transformation, polarization, orientation and migration were provisionally ascribed to interaction between dispersed cells in an environment consisting essentially of populated semi-solid medium bordered by a zone of cell-free medium. As the cells were not usually or necessarily in immediate physical contact, their interactions were effected through the medium. This was presumably achieved by means of soluble substances produced by the cells and liberated into the medium. These diffusible products were probably effective in themselves but interaction between them and the medium should not be excluded.

The primary assumption that the cell interactions described above were due to soluble products of cellular origin implied the secondary corollaries that local and general concentrations of diffusible cell products were proportional to cell number and vitality and that regional differences in population density produced concentration gradients of the soluble factors. In a limited amount of culture medium concentration gradients probably tended to diminish with duration of cultivation while absolute concentrations simultaneously increased.

Two contrasting types of effects upon cell activities were detectable in the "pure" cultures. Degeneration of cells at the centres of dense cultures resulted from the accumulation of harmful substances and/or lack of essential substances. On the other hand, the tumour cells were mutually protective. The predominance of one or other of these effects depended upon the population density which pertained. Three kinds of gradients were distinguished. One of beneficial and protective substances and another of harmful metabolic products were both of cellular origin and diminished centrifugally from centres of population. "Deprivation" gradients due to consumption by the cells of nutrients, etc., diminished centripetally.

It was earlier suggested (Powell, 1957*b*), on the basis of observed correlations between population density, and cell viability and division, respectively, in " pure " cultures of ascites tumour cells, that these functions depended on absolute concentrations of protective factors. Concentration gradients were important to other cell activities.

The migration of cells away from centres of population and therefore of protective factors suggested that the movement was an " escape reaction " from harmful products to more favourable medium. There appeared to be a balance between impelled migration and dependence upon protective factors.

Cell polarity was possibly related to local gradients with effective differences in concentration over distances of the order of a cell diameter. The pole of a cell exposed to the lower intensity of repelling factors became anterior and the one at the higher concentration became posterior. Migration of polarized cells was in the direction of the lower concentration. The radial orientation of marginal polarized cells was explicable by equal sensitivity of the sides of a cell as the presence of cytoplasmic filaments indicated that migration was an active process involving extension of the anterior and retraction of the posterior end of the cell. The general absence of polarity and directed movement of transformed cells surrounded by numerous other cells accorded with these conceptions. In such conditions and the similar ones at the edges of dense masses of cells gradients would at best be ill-defined.

The rate of production of the protective factors by an isolated living tumour cell was less than the rate at which they were lost by it to the medium. The net amount of protective factors available to an isolated cell was therefore less than its requirements. Such a cell, being unable to meet the deficiency by taking up supplies from the medium, could not survive and reproduce. The significance of concentration gradients of protective factors was not apparent in the present experiments but they are important in the growth regulation of ordinary explants. Fischer (1946) believed that intercellular protoplasmic connections were mainly responsible for the organization of tissue cultures. This view is not supported by the observations made in the present work upon dispersed tumour cells.

SUMMARY

The structural changes and behaviour of Ehrlich carcinoma and Sarcoma 37 ascites tumour cells in " pure " cultures in plasma media are described.

The phenomena reported are attributed to cellular interaction mediated by soluble products diffusing from the cells into the medium.

Viability and division of the cultured tumour cells depend on certain minimal concentrations of protective substances of cellular origin.

Cell polarity, migration and orientation of the cells are regulated by concentration gradients caused by the vital activities of the tumour cells.

I am indebted to Mr. G. A. Butcher for his assistance with these researches.

The expenses of this work were defrayed from a block grant by the British Empire Cancer Campaign.

REFERENCES

FISCHER, A.—(1946) ' Biology of Tissue Cells '. London (Cambridge University Press).
KLEIN, E.—(1954) *Cancer Res.*, **14**, 482.—(1955) *Exp. Cell. Res.*, **8**, 213.
LASNITZKI, I.—(1952) *J. Path. Bact.*, **64**, 252.—(1953) *Brit. J. Cancer*, **7**, 288.
POWELL, A. K.—(1957*a*) *Brit. J. Cancer*, **11**, 112.—(1957*b*) *Ibid.*, **11**, 274.
SCHLEICH, A.—(1954) *Cancer Res.*, **14**, 486.—(1956) *Ann. N.Y. Acad. Sci.*, **63**, 849.